# Feature augmentation based on information fusion rectification for few-shot image classification

**DOI:** 10.1038/s41598-023-30398-1

**Published:** 2023-03-03

**Authors:** Hang Wang, Shengzhao Tian, Yan Fu, Junlin Zhou, Jingfa Liu, Duanbing Chen

**Affiliations:** 1 Big Data Research Center, University of Electronic Science and Technology of China, Chengdu, 611731 China; 2 Chengdu Union Big Data Tech. Inc., Chengdu, 610041 China; 3 Guangzhou Key Laboratory of Multilingual Intelligent Processing, Guangdong University of Foreign Studies, Guangzhou, 510006 China; 4 School of Information Science and Technology, Guangdong University of Foreign Studies, Guangzhou, 510006 China

**Keywords:** Computer science, Information technology, Scientific data

## Abstract

In the issue of few-shot image classification, due to lack of sufficient data, directly training the model will lead to overfitting. In order to alleviate this problem, more and more methods focus on non-parametric data augmentation, which uses the information of known data to construct non-parametric normal distribution to expand samples in the support set. However, there are some differences between base class data and new ones, and the distribution of different samples belonging to same class is also different. The sample features generated by the current methods may have some deviations. A new few-shot image classification algorithm is proposed on the basis of information fusion rectification (IFR), which adequately uses the relationship between the data (including the relationship between base class data and new ones, and the relationship between support set and query set in the new class data), to rectify the distribution of support set in the new class data. In the proposed algorithm, feature of support set is expanded through sampling from the rectified normal distribution, so as to augment the data. Compared with other image augmentation algorithms, the experimental results on three few-shot datasets show that the accuracy of the proposed IFR algorithm is improved by 1.84–4.66% on 5-way 1-shot task and 0.99–1.43% on 5-way 5-shot task.

## Introduction

Deep learning has made great achievements in the research of automatic driving^1^, biometric recognition^2^, face recognition^3^, computer-aided diagnosis^4^, and other image recognition researches, but this technology requires large-scale and high-quality labeled data for supervised training. However, in some specific application scenarios, it is difficult to obtain large-scale and high-quality labeled data for cost, privacy or security ethics, etc. The dependence on data seriously hinders the further development of image recognition based on deep learning. Therefore, how to make deep learning models effectively learn and generalize with only a small number of samples and narrow the gap between artificial intelligence and human intelligence has become an urgent problem in deep learning. This problem is called Few-shot learning (FSL), which is also called few-shot image recognition in the field of image recognition. Specifically, the purpose of few-shot learning is to learn a model with good generalization through a few samples, that is, the model can accurately predict other samples of the same category only by learning one or a few samples.

The few-shot learning models are driven by *N*-way *K*-shot learning tasks^5^, where *N* is the number of categories and *K* is the number of labeled samples in each category. Each *N*-way *K*-shot learning task T has a pair of support set *S* and query set *Q*, where $S=\{(x_i,y_i)\}_{i=1}^{N\times K}$ consists of $N\times K$ samples, and $Q=\{(x_i,y_i)\}_{i=1}^{N\times q}$ consists of $N\times q$ samples. The labels of samples in support set are known and that in query set are unknown. The target of each task is to predict the labels for samples in the query set. There are few annotated samples in support set, hence, to train the model to accomplish target *N*-way *K*-shot tasks, it is usually that leverage other classes with massive labeled samples to help the model gain some class-independent capabilities. The dataset composed of the other classes is called base class dataset. Correspondingly, the dataset composed of target *N*-way *K*-shot tasks is called new class dataset. There is no overlap of categories between base class dataset and new class dataset. In recent years, with the continuous development of deep learning, especially the great success of convolutional neural network in computer vision, the researches on few-shot learning have shown a blowout development. According to how the base class dataset is utilized to solve the *N*-way *K*-shot task in new class dataset, the few-shot learning methods can be broadly divided into two categories: data augmentation, metric and meta learning. The following reviews present related work according to these two categories.

### Metric and meta learning

In methods of metric and meta learning, massive *N*-way *K*-shot tasks with similar form (*N* and *K*) but different classes are constructed based on the base class dataset. In these constructed tasks, the labels of samples in query set are known and used as the ground truth to train model with the same paradigm of learning as the target tasks. The model learns meta knowledge that is independent of specific categories. Then, the model is tested on the target *N*-way *K*-shot tasks to make predictions for the query set samples. Specifically, the entire *N*-way *K*-shot construction process is to first randomly sample *N* categories data from dataset, then randomly select *K* samples in each category as the support set, and finally randomly select *q* samples from the remaining samples of each category as query set. Training on massive *N*-way *K*-shot episodic tasks from the base class dataset allows the models to learn some generalized capabilities, such as meta-knowledge or measurements. Then the models are tested on the new class dataset without additional training steps. Methods can be divided into two sub-categories based on the ability that the model is going to learn: metric learning and meta learning.

The core idea of metric learning is to learn a pairwise similarity feature embeddings between support set and query set. Prototypical Network^6^ is used to learn a metric space in which classification can be performed by utilizing the distances between prototype representations of each class and query feature. Prototype Rectification Network (PRN)^7^ performs prototype rectification from the perspective of intra-class and inter-class respectively, so that the prototype can more truly represent the center of the support set samples than Prototypical Network. Covariance Metric Network (CovaMNet)^8^ obtains the covariance statistics of each category through feature extraction module and calculates the distribution consistency between query set and support set to complete the classification. DN4^9^ and Asymmetric Distribution Measure (ADM)^10^ compare the local descriptors between query set and all support set to find the category closest to complete the classification of the query set. Feature Map Reconstruction Network (FRN)^11^ estimates the support set category prototype by the least squares method and then uses Euclidean distance between query set sample and support set category prototype to classify.

The core idea of meta learning is to enable the model to master a learning ability, that is, to learn meta-knowledge that refers to the additional knowledge learned in meta training process such as hyperparameters of model. Latent Embedding Optimization (LEO)^12^ introduces a low-dimensional latent space and updates parameter through inner loops. Synthetic Information Bottleneck (SIB)^13^ is a transductive method that formulates a variational posterior as a function of support and query sets. It also uses a single meta-learner and optimizes the learner by running several synthetic gradient steps on query set. Ensemble of epoch-wise empirical bayes model (E$^3$BM)^14^ is a generic method that learns to combine the epoch-wise base-learners, and to generate task-specific learning rates and combination weights that encourage robust adaptation.

### Data augmentation

In methods of data augmentation, for each *N*-way *K*-shot task, the core idea is to train a classification model directly by using labeled support set and unlabeled query set to assign the correct labels to the query set. The base class dataset is usually used for pre-training of non-specific recognition tasks that make a feature extracting model to learn effective image representations. The backbone of pre-trained model is used as a feature extractor to train a simple classifier on the new class dataset with few annotated samples. In addition, benefiting from massive amounts of annotated samples, the base class dataset can also provide effective base points of class prototypes for new class dataset.In methods to data augmentation, the unlabeled query set in new class dataset is treated as an input for each *N*-way *K*-shot episodic task. The information contained in the unlabeled samples will be used for data augmentation. Since the ultimate goal is to produce label predictions for the query set, using the query set information without knowing the labels does not cause data leakage.

Mangla et al.^15^ proposed a pre-training methods S2M2 on the base class dataset. The backbone of pre-trained model was used as a feature extractor to train a simple classifier on the new class dataset with few annotated samples. The effectiveness of S2M2 has aroused the interest of researchers on how to improve the performance of classifier with an effective feature extractor, statistically significant class prototypes of base classes and few annotated samples of new classes. In this background, many data augmentation methods have been proposed to solve the *N*-way *K*-shot problem in new class dataset. The core idea of data augmentation is to increase training samples ($N \times K$ samples in *S*) and train a robust classifier to predict the samples in *Q* in each episodic task T. The augmented samples can enhance data diversity to alleviate overfitting of classifier model caused by insufficient data. Attributed-Guided Augmentation (AGA)^16^ learns a mapping that allows synthesis of data such that an attribute of a synthesized sample is at a desired value or strength. TriNet^17^ maps sample features to semantic space through encoder and guides the decoder to synthesize new features based on semantic relations between categories in the semantic space. Attribute-Based Synthetic Network (ABS-Net)^18^ constructs a repository of attribute features by an attribute learning process on the auxiliary dataset, then given the attribute description of one class, a probability based sampling strategy is exploited to select some attribute features from the repository to synthesize combined features. Diversity Transfer Network (DTN)^19^ learns to transfer latent diversities from known categories and composite them with support features to generate diverse samples for new categories in feature space. Shrinking and Hallucinating (SH)^20^ generates data through a transferable intra-class transformation between two samples belonging to the same category. Delta-Encoder^21^ extracts transferable intra-class transformation from auxiliary datasets and applies it to new class so as to synthesize new samples. Wang et al.^22^ combine generative model and classification model to realize end-to-end meta-learning training optimization and make the generated images more suitable for classification task. Zhang et al.^23^ utilize object detection algorithm to separate foreground and background of image, and then randomly combine the foreground and background of different images to generate more images. Distribution Calibration (DC)^24^ uses distribution statistics (mean and covariance) of the base class data to calibrate the statistics of new class and extend the features of new class through sampling. Wu et al.^25^ propose to obtain the distribution statistics of new class through the maximum a posteriori (MAP) of base class and extend the features of new class through sampling. Capture Query Distribution (CQD)^26^ uses support set samples to capture the distribution statistics of query set samples and to generate sample features according to statistical information.

Obviously, the two categories of the few-shot learning methods are quite different. The main differences are how to use the base class dataset and whether to train on the new class dataset for specific *N*-way *K*-shot episodic tasks. Despite the huge differences, methods of both two categories had achieved satisfactory and competitive performance. In this report, we focus on data augmentation methods for few-shot learning following above researches. Specifically, the bias between the base and new class data as well as the difference of distributions of different samples belonging to same class may bring some limitations when the augmented features are generated by the current data augmentation methods. Therefore, a new feature augmentation method is proposed in this report on the basis of information fusion rectification (IFR) for few-shot image classification, which makes full use of relationship between datasets. More specifically, first, model will be pre-trained on the base class dataset to obtain the general weight. Then, feature distributions of support set will be rectified utilizing query set and base class prototype obtained by pre-trained model and the features of support set will be expanded by rectified distribution. Finally, a simple classifier such as Logistic Regression, Support Vector Machine and Multi-layer Perceptron will be trained utilizing expanded and original support features. Compared with other data augmentation methods, the experimental results on three few-shot datasets show that the accuracy of the proposed IFR method are improved by 1.84–4.66% on 5-way 1-shot task and 0.99–1.43% on 5-way 5-shot task.

The remainder of this report is organized as follows. The feature augmentation method based on IFR and the few-shot Learning framework with IFR based feature augmentation are introduced in Sect. “Methods”. The experimental results and analysis are presented in Sect. “Results and Discussion”. Conclusions and future work are presented in Sect. “Conclusion”.

## Methods

The core idea of feature augmentation based on information fusion rectification is takes both relationships of the support dataset to the base dataset and the query dataset into account by using cosine similarity to find the most relevant base class prototypes and query features. The information fusion rectification module is proposed to efficiently fuse two kinds of information and rectify the distribution of support dataset. The cosine similarity values are taken as its corresponding weight to make full use of the effective information of base class data and query set. The features generated by IFR are not only closely related to the current few-shot task, but also can make full use of the information from base class dataset.

### Feature augmentation based on information fusion rectification

**Figure 1:**
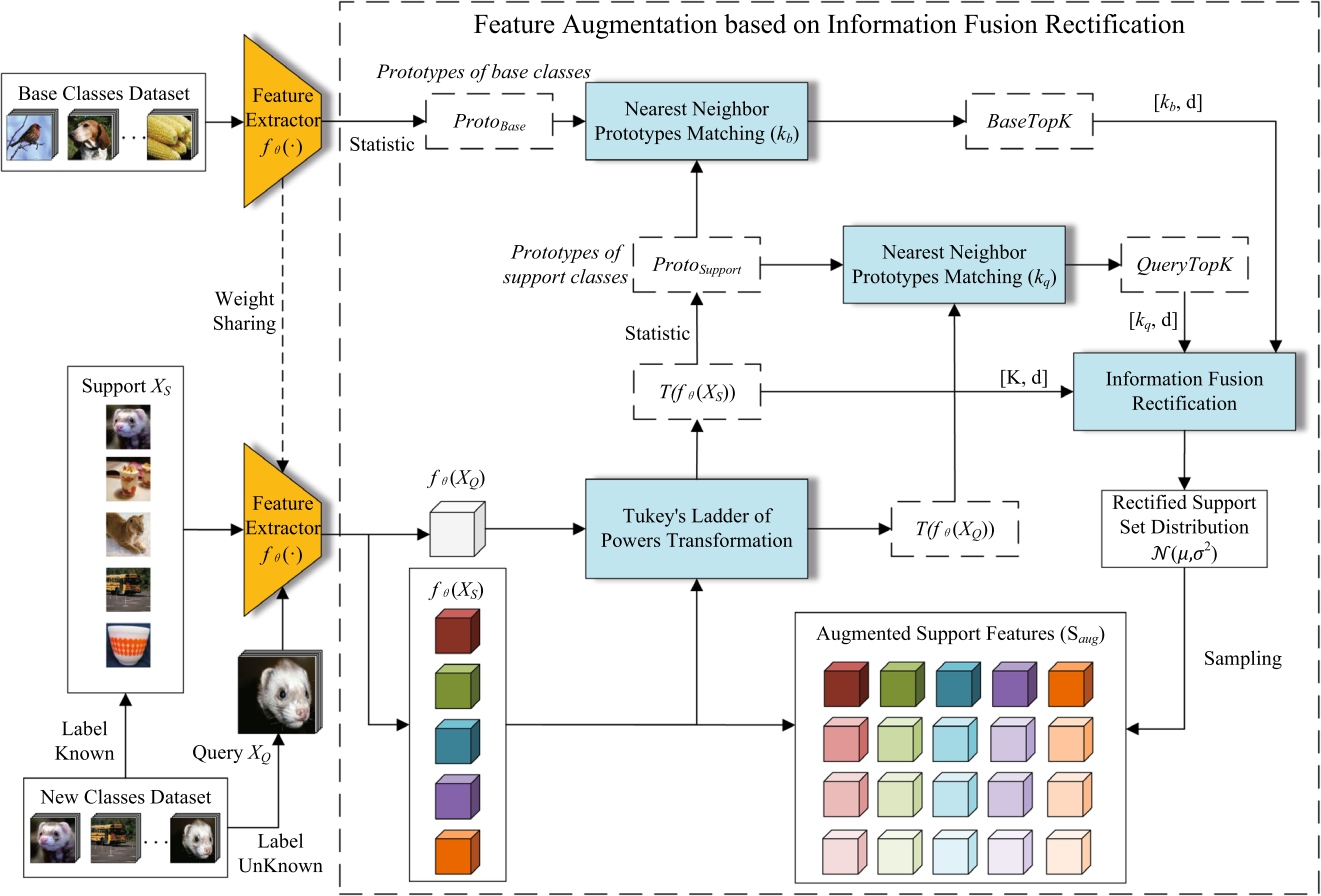
Feature augmentation based on information fusion rectification.

The feature augmentation based on IFR proposed in this study is a data augmentation method in few-shot learning. Therefore, the problem is set under the *N*-way *K*-shot problem. For a given episodic task T, the support set $S=\{(x_i,y_i)\}_{i=1}^{N\times K}$ consists of $N\times K$ samples with known class labels. The query set $Q=\{(x_i,y_i)\}_{i=1}^{N\times q}$ consists of $N\times q$ samples with unknown class labels. The ultimate goal is to predict the class of samples in *Q* by learning in *S* for T. However, there are very few samples in support set. For example, there are only 25 annotated samples in a common setting of 5-way 5-shot. Overfitting will occur if the classifier is trained directly with support sets. The classifier will not be able to effectively predict the labels of the query set samples. The IFR based feature augmentation method is proposed to increase the diversity of training data in *S*. The method is illustrated in Fig. 1.

At the beginning, samples from base class dataset are input into the feature extractor to obtain the corresponding deep features. The prototypes of base classes ($\textbf{proto}_{Base}$) are obtained by taking average of all feature vectors of samples:

1 $$\begin{aligned} \textbf{proto}_i=\frac{\sum _{j=1}^{n_i}f_\theta ({\textbf{x}}_i^j)}{n_i} \in \textbf{proto}_{Base}, \end{aligned}$$

where $\textbf{proto}_i$ is the prototype vector of class *i* in base class, $n_i$ is the number of samples in class *i*,$f_\theta ({\textbf{x}}_i^j)\in {\mathbb {R}}^d$ is *d* dimension feature vector of the *j*th sample of class *i*. The generation of $\textbf{proto}_{Base}$ needs to be executed once as long as the base class dataset does not change.

The IFR based feature augmentation method contains three important module: the Tukey’s ladder of powers transformation module, the nearest neighbor prototypes matching module and the information fusion rectification module. The Tukey’s ladder of powers transformation has proved to be effective in reducing the deviation of the distributions by DC^24^, MAP^25^, CQD^26^. Hence, the Tukey’s ladder of powers transformation is adopted in IFR based feature augmentation method.

For the nearest neighbor prototypes matching module, the similar idea was used in CQD^26^ and DC^24^ . However, only the relationship between the base class prototypes and the prototypes of support dataset was considered in DC^24^ and only the relationship between the query features and the prototypes of support dataset was considered in CQD^26^. Differently, our method takes both relationships into account by using cosine similarity to find the closest base class prototypes and query features. The information fusion rectification module is proposed to efficiently fuse two kinds of information and rectify the distribution of support data. The cosine similarity values are taken as its corresponding weight to make full use of the effective information of base class data and query set. Expanded support features can effectively alleviate overfitting and can improve the accuracy of few-shot image classification.

#### Tukey’s ladder of powers transformation module

For each *N*-way *K*-shot episodic task in new class dataset, first, samples from support set *S* and query set *Q* are input into the feature extractor to obtain the corresponding image features $f_\theta ({S})$ and $f_\theta ({Q})$. Then, the Tukey’s ladder of powers transformation is adopted to reduce the deviation of the distribution. The transformation makes the distribution of deep features close to a Gaussian distribution, which is convenient for subsequent rectification. Specifically, if ${\textbf{x}}$ is an input deep feature vector, the function of Tukey’s ladder of powers transformation can be expressed as

2 $$\begin{aligned} T({\textbf{x}}) = \left\{ {\begin{array}{*{20}{l}} \mathbf {{\textbf{x}}}^\lambda &{}\lambda \ne 0\\ log({\textbf{x}})&{}\lambda = 0 \end{array}} \right. , \end{aligned}$$

where $T({\textbf{x}})$ is the feature vector after transformation, and $\lambda$ is a hyperparameter to adjust the mapping distribution.

For the transformed deep features in support set, because the classes of samples are known, the prototypes of support set ($\textbf{proto}_{Support}$) are obtained by taking average of all feature vectors of samples:

3 $$\begin{aligned} \textbf{proto}_p=\frac{\sum _{j=1}^{K}T(f_\theta ({\textbf{x}}_p^j))}{K} \in \textbf{proto}_{Support}, \end{aligned}$$

where *K* is the number of samples in support class *p*. $\textbf{proto}_p$ is the prototype vector of class *p* in support set. $T(f_\theta ({\textbf{x}}_p^j))\in {\mathbb {R}}^d$ is *d* dimension transformed feature vector of the *j*th sample of class *p* in support set. The transformed deep features in query set $T(f_\theta ({Q}))$ are directly used in following process due to the unknowability of the class labels.

#### Nearest neighbor prototypes matching module

In the next, the nearest neighbor prototypes matching is used to find the close prototype vectors of base class and the close transformed deep features of query set. Specifically, the nearest neighbor prototypes matching is performed twice for each class prototype in $\textbf{proto}_{Support}$.

For a $\textbf{proto}_p\in \textbf{proto}_{Support}$, in the first nearest neighbor prototypes matching, top $k_b$ prototypes that are closest to $\textbf{proto}_p$ are selected from $\textbf{proto}_{Base}$. In the second nearest neighbor prototypes matching, top $k_q$ feature vectors that are closest to $\textbf{proto}_p$ are selected from $T(f_\theta ({Q}))$. Cosine similarity is be considered as the distance measurement in this study. The distances between $\textbf{proto}_p$ and prototypes in $\textbf{proto}_{Base}$ can be calculated by:

4 $$\begin{aligned} disB=\left\{ \frac{(\textbf{proto}_p)^T\textbf{proto}_i}{||\textbf{proto}_p||\cdot ||\textbf{proto}_i||},\textbf{proto}_i\in \textbf{proto}_{Base}\right\} , \end{aligned}$$

The distances between $\textbf{proto}_p$ and transformed deep features in $T(f_\theta ({Q}))$ can be calculated by:

5 $$\begin{aligned} disQ=\left\{ \frac{(\textbf{proto}_p)^TT(f_\theta ({x_i}))}{||\textbf{proto}_p||\cdot ||T(f_\theta ({x_i}))||},T(f_\theta ({x_i}))\in T(f_\theta ({Q}))\right\} , \end{aligned}$$

Finally, *BaseTopK* and *QueryTopK* can be constructed by:

6 $$\begin{aligned} BaseTopK=\{\textbf{proto}_i\times disB_i, i\in topK(disB)\}, \end{aligned}$$

and

7 $$\begin{aligned} QueryTopK=\{T(f_\theta ({x_i}))\times disQ_i, i\in topK(disQ)\}, \end{aligned}$$

where *BaseTopK* is the top $k_b$ base class prototype vectors multiplied by its distance based on *disB* and *QueryTopK* is the top $k_q$ query feature vectors multiplied by its distance based on *disQ*.

#### Information fusion rectification module

In information fusion rectification module, the ultimate goal is to construct a suitable set of Gaussian distributions to generate augmented features. For each class *p* in an *N*-way *K*-shot task, there are base class prototype vectors set *BaseTopK*, query feature vectors set *QueryTopK* and all transformed feature vectors of the sample of class *p* in support set $T(f_\theta ({\textbf{X}}_p))$. These vectors are concatenated to a new feature vectors set. If the dimension of feature vectors are *d*, the *BaseTopK* is a set of feature vectors with dimension $[k_b, d]$, the *QueryTopK* is a set of feature vectors with dimension $[k_q, d]$, and the $T(f_\theta ({\textbf{X}}_p))$ is a set of feature vectors with dimension [*K*, *d*]. Hence, The dimension of concatenated set of feature vectors is $[k_b+k_q+K, d]$. The standard deviation of target distributions to be constructed can be calculated as:

8 $$\begin{aligned} \sigma =\sqrt{\frac{1}{k_b+k_q+K}\sum _{i=1}^{k_b+k_q+K}\left( {\textbf{x}}_i -\frac{1}{k_b+k_q+K}\sum _{j=1}^{k_b+k_q+K}{\textbf{x}}_j\right) ^2}, \end{aligned}$$

where $\sigma \in {\mathbb {R}}^d$ and ${\textbf{x}}_i$ is the *i*th element of d-dimension vectors. Then, Gaussian distributions of support class *p* can be constructed by standard deviation $\sigma$ and query feature vectors set *QueryTopK*:

9 $$\begin{aligned} \left\{ {\mathcal {N}}_p(\frac{QueryTopK[i]}{disQ_i},\sigma )\right\} _{i=1}^{k_q}. \end{aligned}$$

By fusing the information of base classes (*BaseTopK*) and query samples (*QueryTopK*), the distribution of support samples ($T(f_\theta ({\textbf{X}}_p))$) are rectified to a set of Gaussian distributions. For each class *p* in support set, there are $k_q$ rectified distributions. Finally, *m* samples from each distribution are sampled to expand features of the corresponding support class. The overall process of IFR based feature augmentation method is shown in Algorithm 1.
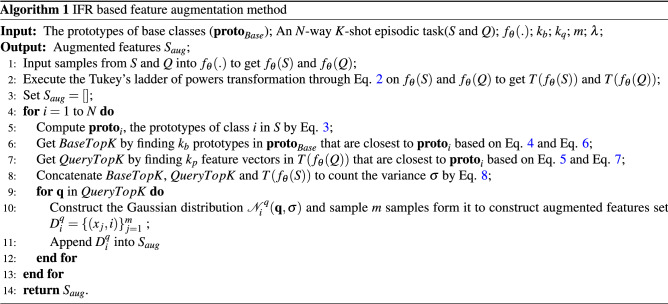


### Few-shot learning framework with IFR based feature augmentation

**Figure 2:**
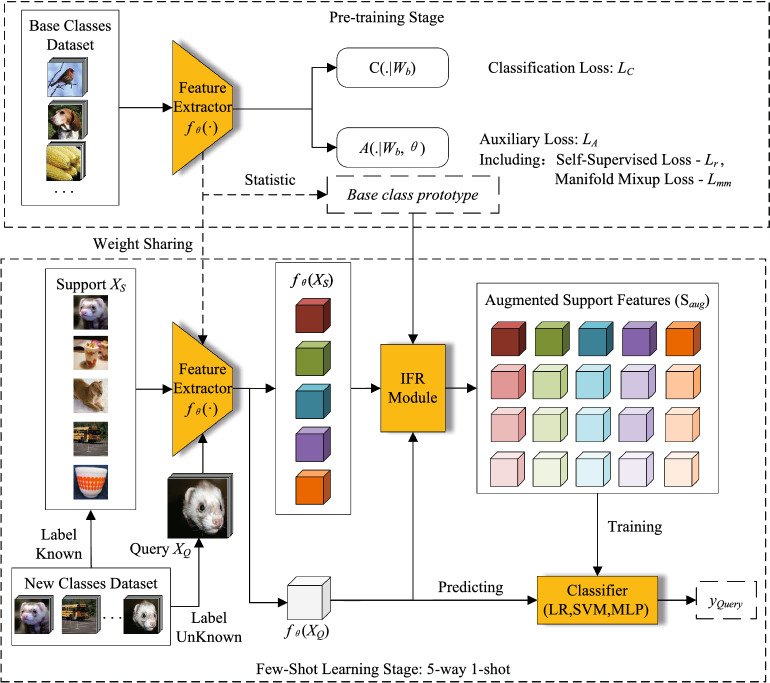
The framework of few-shot learning with IFR based feature augmentation (5-way 1-shot).

The framework of few-shot learning with IFR based feature augmentation is shown in Fig. 2. The framework of few-shot learning with IFR based feature augmentation includes two main stages: pre-training stage and few-shot learning stage.

#### Pre-training stage

As shown in Fig. 2, in pre-training stage, a feature extractor $f_\theta (\cdot )$ is trained by prediction tasks on the base class dataset. Specifically, WideResNet is selected as the feature extractor in this study like most other methods. The backbone network of pre-trained classification model is retained as the feature extractor. In addition to the common classification loss, auxiliary losses such as self-supervised loss^27^ and manifold mixup loss^15^ are also used to provide enough decision boundaries among classes to make the model generalize to new class. These auxiliary losses have been shown to have better pre-training effectiveness in studies such as S2M2^15^.

For self-supervised training, image rotation prediction^27^ is used as a unsupervised pre-training task in this study. The rotated image is used as the input of model, and the model learned to predict how much degree the input image has been rotated. The purpose of rotation prediction is to make the model to understand the position, type and attitude of image objects and make the backbone network of the model to extract more characteristic features. Four rotation angles are set in the first to process the image. Each input image *x* is rotated by *r* degrees, in which $r \in C_R=\{0,90,180,270\}$. The rotated image is input into the model to extract its deep features, and finally the rotated angle is predicted by model.

Manifold mixup^15^ is an image augmentation method. The deep features of different images are linearly combined to expand the training samples and improve the generalization ability of the model. Given two image samples and its labels $(x_i,y_i)$ and $(x_j,y_j)$, the manifold mixup image and label are defined as:

10 $$\begin{aligned} \begin{aligned} x_{mm} = \gamma f_\theta ^l(x_i) + (1-\gamma )f_\theta ^l(x_j), \\ y_{mm} = \gamma y_i + (1-\gamma )y_j, \end{aligned} \end{aligned}$$

where $x_{mm}$ is mixup feature and $y_{mm}$ is mixup label. $f_\theta ^l(x_i)$ and $f_\theta ^l(x_j)$ are the output features of model $f_\theta$ at layer *l* for input images $x_i$ and $x_j$. $\gamma$ is a hyperparameter to control the degree of mixup. The model trained by manifold mixup can capture more advanced information in different layers that can improve the generalization ability of the model.

Overall, there are three losses in pre-training stage including classification loss, rotation self-supervised loss and manifold mixup loss. ${\mathcal {L}}_{C}(x^r, y)$ denotes the cross-entropy loss of model with a batch of rotated samples $x^r$ and the corresponding labels *y*. ${\mathcal {L}}_{r}(x^r, r)$ denotes the cross-entropy loss of model with a batch of rotated samples $x^r$ and the corresponding rotated degree labels *r*. ${\mathcal {L}}_{mm}(x_{mm}, y_{mm})$ denotes the cross-entropy loss of model with a batch of mixup samples $x_{mm}$ and the corresponding mixup labels $y_{mm}$.

The whole pre-training process is divided into two steps. First, the model is trained using rotation self-supervised loss and classification loss. Then, the model is trained for fine-tuning by all three losses. The process of pre-training stage is shown in Algorithm 2.
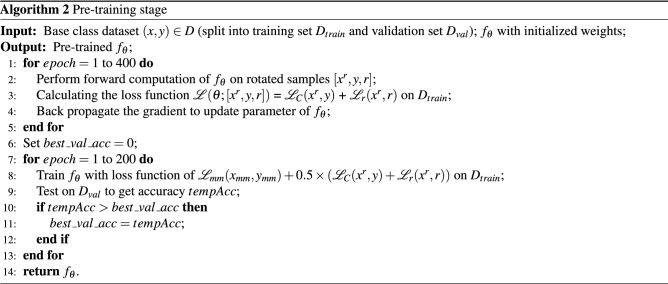


#### Few-shot learning stage

In few-shot learning stage, an *N*-way *K*-shot episodic task is constructed from new class dataset with support set *S* and query set *Q*. First, *S* and *Q* are input into the feature extractor to obtain the corresponding image features $f_\theta ({S})$ and $f_\theta ({Q})$. Then, IFR module proposed in this report is used to obtain the augmented support features $S_{aug}$. Finally, a simple machine learning classification model such as Logistic Regression, Support Vector Machine or Multi-layer Perceptron is trained with the augmented support features, and the query set are predicted directly through the model after training. The process of few-shot learning stage is shown in Algorithm 3.
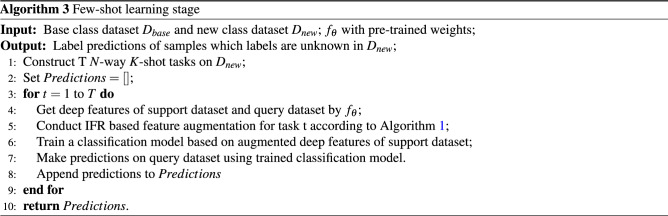


## Results and discussion

The experiments are designed based on following two aspects. First, comparative experiments are conducted to evaluate the proposed method and other benchmark methods on three datasets. Second, different variants of components in feature augmentation method and different hyperparameters of proposed method are explored as ablation experiments to prove that the proposed method can achieve the best performance.

### Datasets

In order to evaluate the performance of the proposed IFR based feature augmentation, three popular datasets: miniImageNet, tieredImageNet and CUB, are used in the experiments.

The miniImageNet dataset is a subset of the ImageNet. It contains 100 classes with 600 samples per class. The size of image is not fixed and is resized to $84\times 84$ in this report. The dataset is split into 64 base classes, 16 validation classes and 20 new classes.

The tieredImageNet dataset is also a subset of the ImageNet. It distinguishes classes into broader categories corresponding to higher-level nodes in the ImageNet, so that all of the training classes are sufficiently distinct from the testing classes. And its training, validation, and testing datasets have no semantic relationship and can be viewed as a cross-hierarchical dataset. It contains 779,165 images of 608 classes. The size of the image is not fixed and is resized to $84\times 84$ in this report. The dataset is split into 351 base classes, 97 validation classes and 160 new classes.

The CUB dataset is a bird dataset that is often used as a benchmark for fine-grained visual categorization task. It contains 11,788 images of 200 classes, and the size of image is not fixed and is resized to $32\times 32$ in this report. The dataset is split into 100 base classes, 50 validation classes and 50 new classes.

### Experimental setting details

In pre-training stage, WideResNet is used to pre-train with the same setting as in S2M2^15^, except that all activation functions in WideResNet are set as ReLU to ensure the inputs of Tukey’s ladder of powers transformation are valid.

In few-shot learning stage, standard 5-way 1-shot and 5-way 5-shot episodic tasks are considered with 15 query images in each task. Particularly, hyperparameters of IFR based feature augmentation are set as $\lambda =0.5, k_b=2, k_q=6$, $m=1$, and $d=640$. Logistic regression (LR), support vector machine (SVM) and multi-layer perceptron (MLP) are adopted as classification models in few-shot learning stage. For LR and SVM, training epoch is set as 1,000 and the rest of parameters are default. For MLP, random$\_$state=123, training epoch=500, hidden layer=2 with corresponding neurons being 128 and 64 respectively, and the rest of parameters are default. We trained all classification models from scratch and used the Adam optimizer with 0.001 learning rate. Finally, 10,000 tasks are randomly generated from the new classes and top-1 average accuracy as well as 95% confidence intervals for all datasets are reported.

### Comparative experiments

In the comparative experiments, the results of 5-way 1-shot and 5-way 5-shot classification tasks are compared with related augmentation methods and other state-of-the-art meta learning methods, including DC^24^, MAP^25^, CQD^26^, LEO^12^, SIB^13^, E$^3$BM^14^, S2M2^15^. For the sake of comparability, the feature extractor of each method is set as WideResNet.

The comparison results are shown in Table 1. Despite the huge differences, meta learning methods (LEO^12^, SIB^13^, and E$^3$BM^14^ ) and data augmentation methods (S2M2^15^, DC^24^, MAP^25^, CQD^26^ and proposed IFR) had achieved comparable performance. It can be seen that IFR+LR achieves the highest accuracy on all datasets. Specifically, on miniImageNet, our methods achieved accuracy of 73.40% and 85.29% for 5-way 1-shot and 5-way 5-shot tasks respectively, and improves 2.00% (E$^3$BM^14^) and 0.99% (MAP^25^) when compared with the best accuracy of other comparison methods. On tieredImageNet, our methods achieved accuracy of 80.69% and 89.77% for 5-way 1-shot and 5-way 5-shot tasks respectively, and improves 4.66% (MAP^25^) and 1.18% (S2M2^15^) when compared with the best accuracy of other comparison methods. On CUB, because the LEO^12^, SIB^13^, and E$^3$BM^14^ are not tested on CUB dataset in their original paper, we does not make comparisons for these three methods on CUB. Compared with the best accuracy of other comparison methods on CUB, our methods achieved accuracy of 85.08% and 92.28% for 5-way 1-shot and 5-way 5-shot tasks respectively, and improves 1.84% (CQD^26^) and 1.43% (S2M2^15^). In brief, compared with relevant data augmentation methods (S2M2^15^, DC^24^, MAP^25^, CQD^26^), the experimental results on three few-shot datasets show that the accuracy of the proposed IFR method is improved by 1.84–4.66% on 5-way 1-shot task and 0.99–1.43% on 5-way 5-shot task.

Our method can not only effectively alleviate domain shift between base class and new class, but also may reflect its “true” distribution through the rectified support distribution. It is evidenced by the large performance gain on 5-way 1-shot task in tieredImageNet (4.66%). There is no semantic correlation between base class and new class since tieredImageNet is divided on the basis of hierarchical information. Our method performs well on tieredImageNet and proves that it can be better applied to the task of cross-hierarchical classification.

**Table 1:** 5-way 1-shot and 5-way 5-shot classification accuracy(%) on miniImanegNet, tieredImageNet and CUB.

Methods	miniImageNet		tieredImageNet		CUB	
	5-way 1-shot	5-way 5-shot	5-way 1-shot	5-way 5-shot	5-way 1-shot	5-way 5-shot
LEO^12^	$61.76\pm 0.08$	$77.59\pm 0.12$	$66.33\pm 0.05$	$82.06\pm 0.08$	–	–
SIB^13^	$70.00\pm 0.60$	$79.20\pm 0.40$	72.9	82.8	–	–
E$^3$BM^14^	$71.40\pm 0.50$	$81.20\pm 0.40$	$75.6\pm 0.60$	$84.30\pm 0.40$	–	–
S2M2^15^	$64.93\pm 0.18$	$83.18\pm 0.11$	$73.71\pm 0.22$	$88.59\pm 0.14$	$80.06\pm 0.14$	$90.85\pm 0.44$
DC^24^+LR	$68.10\pm 0.20*$	$83.56\pm 0.14*$	$73.97\pm 0.22*$	$88.39\pm 0.14*$	$79.98\pm 0.21*$	$90.57\pm 0.11*$
DC^24^+SVM	$67.83\pm 0.20*$	$82.25\pm 0.14*$	$72.51\pm 0.23*$	$87.40\pm 0.15*$	$79.90\pm 0.21*$	$90.60\pm 0.11*$
DC^24^+MLP	$67.21\pm 0.21*$	$82.93\pm 0.14*$	$72.75\pm 0.22*$	$83.49\pm 0.19*$	$79.40\pm 0.21*$	$90.30\pm 0.11*$
MAP^25^	$68.48\pm 0.38$	$84.30\pm 0.42$	$76.03\pm 0.08$	$87.26\pm 0.72$	$78.18\pm 0.67$	$89.55\pm 0.36$
CQD^26^+LR	$70.73\pm 0.22*$	$83.33\pm 0.14*$	$71.64\pm 0.24*$	$84.23\pm 0.16*$	$83.00\pm 0.20*$	$90.50\pm 0.11*$
CQD^26^+SVM	$71.14\pm 0.22*$	$83.29\pm 0.14*$	$71.58\pm 0.24*$	$83.92\pm 0.16*$	$83.24\pm 0.20*$	$90.17\pm 0.11*$
CQD^26^+MLP	$70.13\pm 0.22*$	$82.71\pm 0.14*$	$70.58\pm 0.24*$	$83.34\pm 0.16*$	$82.67\pm 0.20*$	$90.23\pm 0.11*$
IFR+LR(Ours)	$\mathbf {73.40\pm 0.22}$	$\mathbf {85.29\pm 0.13}$	$\mathbf {80.69\pm 0.23}$	$\mathbf {89.77\pm 0.13}$	$\mathbf {85.08\pm 0.20}$	$\mathbf {92.28\pm 0.10}$
IFR+SVM(Ours)	$70.97\pm 0.22$	$80.82\pm 0.14$	$77.42\pm 0.23$	$85.74\pm 0.14$	$84.77\pm 0.20$	$90.85\pm 0.10$
IFR+MLP(Ours)	$72.11\pm 0.22$	$84.24\pm 0.13$	$79.47\pm 0.23$	$88.84\pm 0.13$	$83.61\pm 0.20$	$91.29\pm 0.10$

* indicates the result is reported by our reimplementation.

± indicates 95% confidence interval.

The numbers in bold indicates the best performance.

### Ablation experiments

In the ablation experiments, different variants of components in feature augmentation method and different hyperparameters of proposed method are explored as ablation experiments to prove that the proposed method can achieve the best performance.

**Figure 3:**
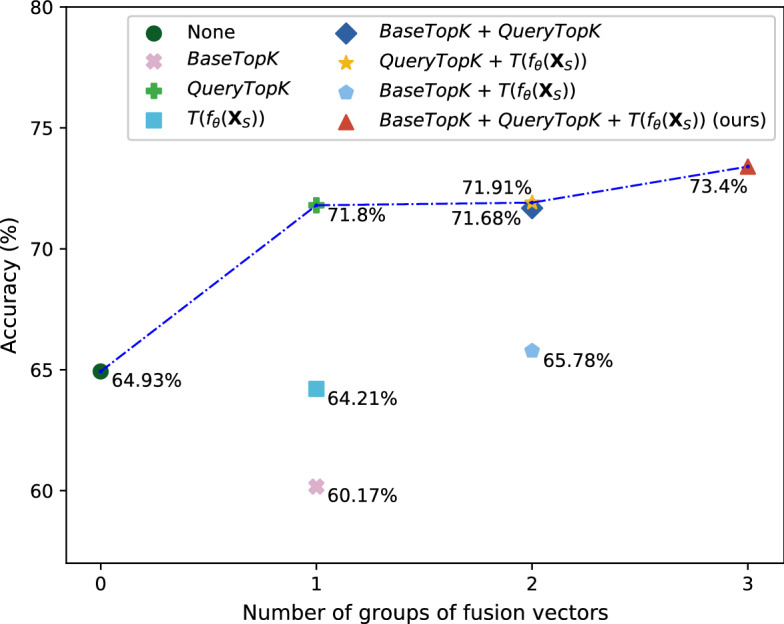
Experimental results of different fusion information on 5-way 1-shot task of miniImageNet dataset. The X-axis coordinate represents the number of groups of fusion vectors, and the Y-axis represents the accuracy of the LR model. Each shape point represents a fusion combination. The number near the shape points represents the accuracy of the LR model. Blue dashed polylines connect the models with the highest accuracy for each number of groups of fusion vectors.

**Figure 4:**
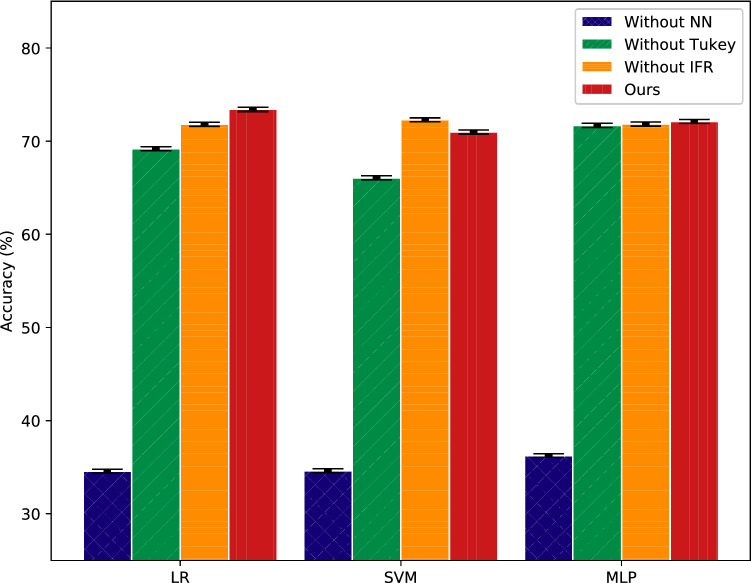
Experimental results of different modules used in IFR based feature augmentation method on 5-way 1-shot task of miniImageNet dataset. The X-axis coordinate represents three classification models, and the Y-axis represents the accuracy of each model. “Without NN” represents that the nearest neighbor prototypes matching module is instead by random select. “Without Tukey” represents that the Tukey’s ladder of powers transformation module is not used in IFR based feature augmentation method. “Without IFR” represents that only query feature vectors set *QueryTopK* is used in feature augmentation.

#### Exploration of different fusion information

In proposed method, the information is fused from three sets of vectors including base class prototype vectors set *BaseTopK*, query feature vectors set *QueryTopK* and all transformed feature vectors $T(f_\theta ({\textbf{X}}_S))$ of support dataset. An ablation experiment is conducted with different combinations of vectors sets. IFR+LR model is taken as example to analyze under the setting of miniImageNet 5-way 1-shot since IFR+LR model has the highest accuracy among all the models. The experimental results are presented in Fig. 3.

Eight combinations are compared in this study. “None” represents the case where none of augmentation methods are conducted. The experimental results show that the information combination proposed in this study can obtain the maximum performance gain. Compared with the best accuracy of single vector and two groups of fusion vectors, the accuracy of model which takes three groups of fusion vectors improves 1.6% and 1.49% respectively, and improves 8.47% from model without any augmentation methods.

In addition, with the increase number of groups of fusion vectors, the blue dashed polylines has a positive slope. The trends of the dashed polylines indicate that in data augmentation, fusion more information by appropriate fusion methods can improve the performance compared with fusion less information or fusion by inappropriate methods. The experimental results proved that the performance gains of proposed method comes from the information fusion and rectification.

#### Exploration of different modules

In proposed method, there are three significant modules: the Tukey’s ladder of powers transformation module and the nearest neighbor prototypes matching module, and the information fusion rectification module. In order to understand their importance, an ablation experiment is conducted under the setting of miniImageNet 5-way 1-shot task. The experimental results are presented in Fig. 4.

Four variations are compared in this study. “Without NN” represents that the nearest neighbor prototypes matching module is instead by random select. “Without Tukey” represents that the Tukey’s ladder of powers transformation module is not used in IFR based feature augmentation method. “Without IFR” represents that only query feature vectors set *QueryTopK* is used in feature augmentation. “Ours” represents training with the proposed IFR based feature augmentation method. The experimental results show that the nearest neighbor prototypes matching module are the most important module in proposed method. This module ensures that the prototypes and feature vectors used for augmentation are related to the task scenario. It effectively avoids to generate unrelated features to the task. Besides, the Tukey’s ladder of powers transformation module also brings some performance improvements. Using the information fusion rectification module achieve the best performance on LR classifier and small performance improvements on MLP classifier. However, on SVM classifier, using the information fusion rectification module do not bring performance gain. It is consistent with the results of competitive experiments.

#### Exploration of different hyperparameters

Since there are three hyperparameters in the information fusion rectification module: $k_b$, $k_q$ and *m*, different parameters may have different influences on the experimental results. Take IFR+LR as example to analyze the influence of hyperparameters on the model under the setting of miniImageNet 5-way 1-shot since IFR+LR model has the highest accuracy among all the models.

**Figure 5:**
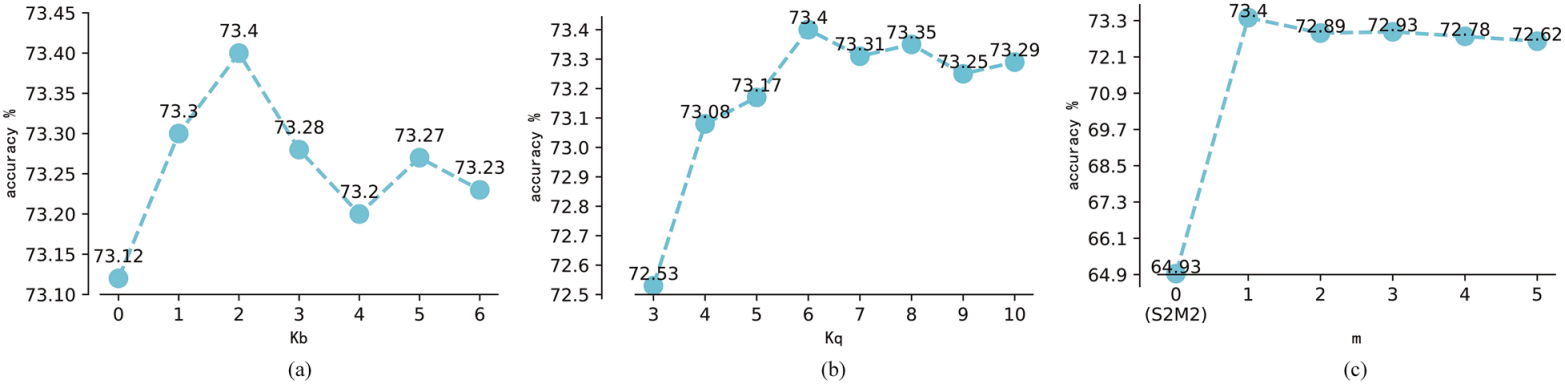
The effect of hyperparameter $k_b$, $k_q$ and *m* on miniImageNet with 5-way 1-shot setting. (**a**) The effect of hyperparameter $k_b$. (**b**) The effect of hyperparameter $k_q$. (**c**) The effect of hyperparameter *m*.

The effect of $k_b$ under $k_q=6$ and $m=1$ is shown in Fig. 5a. When $k_b$ is 2, IFR+LR achieves the best accuracy. With the increase of $k_b$, the accuracy of IFR+LR always remains above 73.2%. Although there are some fluctuations, the value will not bring much change to the classification result, that is, $k_b$ has little effect on the model but is useful.

Experimental results on different value of $k_q$ under $k_b=2$ and $m=1$ are shown in Fig. 5b. The value of $k_q$ is ranges from 1 to 15 in this study since there are only 15 query samples per class in a 5-way 1-shot setting. When $k_q$=6, IFR+LR has the best classification accuracy. If $k_q$ is larger than 6, the classification accuracy always remains above 73.25% with small fluctuation. Actually, if $k_q$ is too small, it is difficult to estimate the distribution accurately since few query features are found, and if $k_q$ is too large, some noises may be introduced since the query features and support prototype might belong to different class.

Experimental results on different value of *m* under $k_b=2$ and $k_q=6$ are shown in Fig. 5c. When *m* is 1, IFR+LR has the best classification accuracy. And the accuracy gradually decreases while *m* increasing. So, it is necessary to generate new samples but the quantity should not be too much. The mean of the rectified distribution of each support class is the first $k_q$ query features closest to the support prototype, the found query features and the support prototype may not belong to same category. The features generated by the rectified distribution may have some deviations. As the number of generated samples *m* continues to increase, the deviation of the generated samples may become larger and larger, resulting in accuracy decreasing. For this reason, a smaller *m* should be selected. If $m=0$, the proposed model in this report turns to S2M2^15^, which directly uses the logistic regression to train the support set and to predict query set, without any data augmentation strategy.

## Conclusion

This study focus on data augmentation methods for few-shot learning following previous studies. A feature augmentation method is proposed in this paper based on information fusion rectification (IFR) for few-shot image classification, which makes full use of relationship between datasets. The relevant information between the base and new class data as well as the support dataset and query dataset is fully utilized in proposed IFR module. The features generated by IFR are not only closely related to the current few-shot task, but also can make full use of the information from base class dataset. Compared with other data augmentation methods, the experimental results on three few-shot datasets show that the accuracy of the proposed IFR method are improved by 1.84–4.66% on 5-way 1-shot task and 0.99–1.43% on 5-way 5-shot task. The results of ablation experiments proved the effectiveness of the proposed method and revealed the influence of hyperparameters of proposed method.

In a future study, we will carry out research on diverse ways of expressing information (not just class prototypes and feature vectors), different fusion approaches, and different ways of utilizing fused information that can improve the performance of few-shot image classification.

## Data Availability

Our data is actually open source data, and it is a dataset commonly used in few-shot image classification. This dataset can be obtained on Kaggle. The specific address is as follows: For miniImageNet, we can get from: https://www.kaggle.com/datasets/arjunashok33/miniimagenet. For tieredImageNet, we can get from: https://www.kaggle.com/datasets/arjun2000ashok/tieredimagenet. For CUB, we can get from: https://www.kaggle.com/datasets/veeralakrishna/200-bird-species-with-11788-images.
